# Aneurysmal subarachnoid haemorrhage from a neuroimaging perspective

**DOI:** 10.1186/s13054-014-0557-2

**Published:** 2014-11-13

**Authors:** Airton Leonardo de Oliveira Manoel, Ann Mansur, Amanda Murphy, David Turkel-Parrella, Matt Macdonald, R Loch Macdonald, Walter Montanera, Thomas R Marotta, Aditya Bharatha, Khaled Effendi, Tom A Schweizer

**Affiliations:** Trauma & Neurosurgery Intensive Care, Department of Medical Imaging, 3-141 CC, St Michael’s Hospital, University of Toronto, 30 Bond Street, Toronto, ON M5B 1 W8 Canada; Medical Imaging, Interventional Neuroradiology, St Michael’s Hospital, University of Toronto, 30 Bond Street, Toronto, ON M5B 1 W8 Canada; Keenan Research Centre of the Li Ka Shing Knowledge Institute of St Michael’s Hospital, 30 Bond Street, Toronto, ON M5B 1 W8 Canada; Medical Imaging, University of Toronto, 30 Bond Street, Toronto, ON M5B 1 W8 Canada; Division of Neurosurgery, St Michael’s Hospital, University of Toronto, 30 Bond Street, Toronto, ON M5B 1 W8 Canada; Faculty of Medicine, University of Toronto, 30 Bond Street, Toronto, ON M5B 1 W8 Canada; Neuroscience Research Program, St Michael’s Hospital, University of Toronto, 30 Bond Street, Toronto, ON M5B 1 W8 Canada

## Abstract

Neuroimaging is a key element in the management of patients suffering from subarachnoid haemorrhage (SAH). In this article, we review the current literature to provide a summary of the existing neuroimaging methods available in clinical practice. Noncontrast computed tomography is highly sensitive in detecting subarachnoid blood, especially within 6 hours of haemorrhage. However, lumbar puncture should follow a negative noncontrast computed tomography scan in patients with symptoms suspicious of SAH. Computed tomography angiography is slowly replacing digital subtraction angiography as the first-line technique for the diagnosis and treatment planning of cerebral aneurysms, but digital subtraction angiography is still required in patients with diffuse SAH and negative initial computed tomography angiography. Delayed cerebral ischaemia is a common and serious complication after SAH. The modern concept of delayed cerebral ischaemia monitoring is shifting from modalities that measure vessel diameter to techniques focusing on brain perfusion. Lastly, evolving modalities applied to assess cerebral physiological, functional and cognitive sequelae after SAH, such as functional magnetic resonance imaging or positron emission tomography, are discussed. These new techniques may have the advantage over structural modalities due to their ability to assess brain physiology and function in real time. However, their use remains mainly experimental and the literature supporting their practice is still scarce.

**Electronic supplementary material:**

The online version of this article (doi:10.1186/s13054-014-0557-2) contains supplementary material, which is available to authorized users.

## Introduction

Aneurysmal subarachnoid haemorrhage (SAH) is caused by the rupture of an intracranial aneurysm, which leads to the extravasation of blood under high pressure into the subarachnoid space. SAH triggers a cascade of events that can result in death or severe disability. Poor outcomes are not uncommon, and are typically due to early brain injury associated with the initial haemorrhage or are secondary to delayed cerebral ischaemia (DCI) and cerebral infarction. This review will focus on the clinical applicability of neuroradiological tools applied to the diagnosis and management of SAH, angiographic cerebral vasospasm (ACV), and DCI.

## Search strategy and selection criteria

A PubMed search for articles published in English until April 2014 was performed using the terms ‘subarachnoid hemorrhage [All Fields] OR subarachnoid haemorrhage [All Fields] OR cerebral aneurysm [All Fields] OR vasospasm [All Fields] OR cerebral vasospasm [All Fields] OR delayed cerebral ischemia [All Fields]’, which returned 84,357 articles (Additional file 1: Table S1). These articles were further distilled via prospective and retrospective studies, review articles, and meta-analyses addressing imaging features of SAH, and their reference lists were also searched. The articles judged to be clinically relevant by the authors were selected. The methodological quality of the meta-analyses reviewed was assessed by the AMSTAR tool (Additional file 1: Table S2) [1]. Additionally, the references from the authors’ personal database were retrieved and also used as a key source for this review. Lastly, the most recent guidelines on the management of SAH and their reference lists were also consulted and used to provide some of the clinical recommendations [2–4].

## Diagnosis of subarachnoid haemorrhage

Headache is a common complaint, accounting for 2% of emergency department (ED) visits [5]. Although SAH represents a very small percentage of those patients admitted to the ED, it is a life-threatening condition that needs to be recognised and treated immediately. Failure to diagnose and treat SAH can lead to dire complications including aneurysm rebleeding, cerebral infarction, and ultimately death [6].

Misdiagnosis, defined as the failure to correctly recognise SAH at first contact between a patient and a medical professional, continues to occur and is associated with increased mortality and morbidity. In a large cohort of 482 SAH patients admitted to a tertiary university hospital, 12% of the cases were misdiagnosed according to the above definition [6]. The likelihood of a missed diagnosis was higher in patients with normal mental status because their symptoms could be misattributed to conditions such as migraine or tension headache, and less commonly to a viral syndrome, musculoskeletal pain, sinusitis, and hypertension [6]. Hence, it is not only imperative to have timely diagnosis, but also to employ the most appropriate and sensitive diagnostic modalities at first consultation in the ED.

The most common diagnostic error is the lack of noncontrast computed tomography (NCCT) ordered by the ED physician [6]. NCCT remains the first-line method for the diagnosis of SAH [3]. Evolving technology has rapidly improved the new generations of multidetector computed tomography (CT) scanners, making this equipment faster, less prone to motion artefacts, of better resolution, and possessing more accurate tools. The combination of this advanced technology with cutting-edge post-acquisition image processing software makes CT scanners more sensitive to detect subarachnoid blood. Additionally, CT is also highly available and affordable, making it the ideal screening modality for patients with suspected SAH.

In a retrospective study using a four-slice, four-detector CT scanner, Byyny and colleagues identified 149 patients with spontaneous SAH who presented to an academic tertiary care ED from 2001 to 2004 [7]. The sensitivity of NCCT for SAH was found to range from 90 to 95%, causing the authors to conclude that NCCT has high sensitivity but should not be applied as a sole diagnostic modality in the diagnosis of SAH. Moreover, NCCT sensitivity decreases with time since haemorrhage; it has close to 100% sensitivity within 12 hours, 93% within 1 day, and less than 60% within 1 week of haemorrhage [8].

A prospective, multicenter study included 11 tertiary EDs from 2000 to 2009 [9]. Using a third-generation CT scanner and imaging interpretation by an experienced neuroradiologist, the group enrolled 3,132 patients who were complaining of having ‘the worst headache of their lives’, of which 240 (7.7%) had SAH. Although the overall sensitivity for SAH was only 92.9% (95% confidence interval (CI), 89.0 to 95.5%), all patients suffering from SAH were identified in the subgroup of 953 patients who were scanned within 6 hours of headache onset, thereby yielding sensitivity of 100% (95% CI, 97.0 to 100.0%), specificity of 100% (95% CI, 99.5 to 100%), negative predictive value of 100% (95% CI, 99.5 to 100%) and positive predictive value of 100% (95% CI, 96.9 to 100%). Additionally, in a retrospective study conducted in an academic Level 1 trauma centre, among the 177 patients who presented to the ED with headache and were scanned with a fifth-generation CT scanner followed by a lumbar puncture (LP), no patients with negative NCCT results were diagnosed with SAH on LP [10].

**Figure 1:**
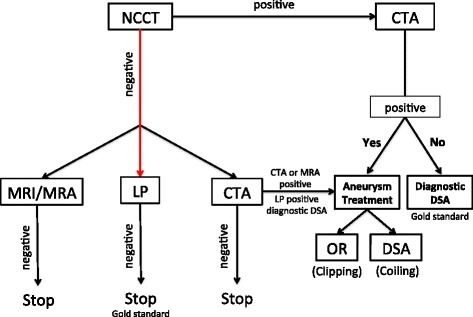
**Diagnostic approach for subarachnoid haemorrhage in patients presenting with more than 6 hours of headache onset.** CTA, computed tomography angiography; DSA, digital subtraction angiography; LP, lumbar puncture; MRA, magnetic resonance angiography; MRI, magnetic resonance imaging; NCCT, noncontrast computed tomography; OR, operating room.

Three possible pathways for the diagnosis of SAH have been described (Figure 1). Although the most invasive, NCCT followed by LP is the most validated pathway to rule out SAH [11]. NCCT combined with LP for patients with high clinical suspicion of SAH have close to 100% diagnostic sensitivity (95% CI, 93.1 to 99.4%) [10,12]. Ideally, cerebrospinal fluid analysis should be performed using spectrophotometry, which has the highest diagnostic accuracy among all cerebrospinal fluid analytical methods; however, this technique is not widely available [13].

The second described approach is the use of NCCT followed by computed tomography angiography (CTA). Some studies showed that negative CTA can rule out aneurysmal SAH in patients with severe headache and negative NCCT or perimesencephalic haemorrhage pattern [14,15]. The main disadvantages of this approach are the need for iodinated contrast and its potential for anaphylactic reaction and nephrotoxicity, but most importantly exposure to radiation.

The third approach is the use of magnetic resonance imaging (MRI) combined with magnetic resonance angiography (MRA). The fluid-attenuated inversion recovery MRI sequence is comparable with NCCT in detecting acute SAH, and is clearly superior for subacute and chronic haemorrhage [16]. MRI is also a powerful tool in the differential diagnosis of intracranial pathologies, including tumours, inflammatory, and infectious processes.

In conclusion, negative NCCT might be enough to rule out SAH in patients presenting within 6 hours of headache onset. Patients presenting beyond the 6-hour mark require additional diagnostic testing if NCCT is negative [3,4].

## Diagnosis of cerebral aneurysms

Sensitivity and specificity for the detection of aneurysms >3 mm using CTA currently approach 100%, and are comparable with digital subtraction angiography (DSA) [17–20]. Conventional CTA might be less sensitive and accurate than DSA for aneurysms <3 mm. However, recent data showed that CTA performed in a 64-row multislice scanner (sensitivity, specificity, and accuracy 96.3%, 100%, and 98.5%, respectively) [21] and subtraction CTA performed in a 320-detector row scanner (sensitivity, specificity, and accuracy 81.8%, 100%, and 93.3%, respectively) are highly accurate techniques to detect even aneurysms <3 mm [22].

The high spatial and temporal resolution of DSA established this modality as the gold standard for cerebral aneurysm detection. However, DSA is invasive, costly, time-consuming, and demands expertise, limiting its use to a certain pool of medical professionals [23]. CTA may serve as an excellent alternative [2]. Two recent high-quality meta-analyses (Additional file 1: Table S2) [19,20] found CTA to be highly accurate (97.2% sensitivity and 97.9% specificity) in diagnosing cerebral aneurysms when compared with DSA.

**Figure 2:**
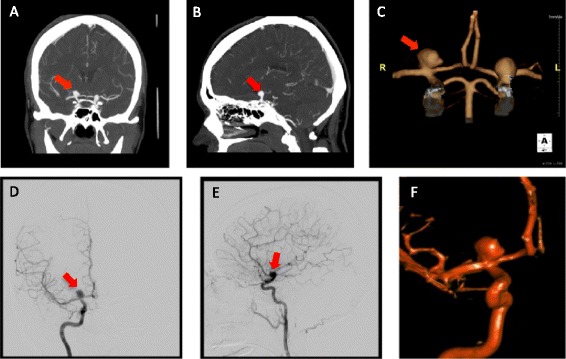
**Computed tomography angiography and digital subtraction angiography. (A)** to **(C)** Computed tomography angiography. **(D)** to **(F)** Digital subtraction angiography. Red arrow, right terminal internal carotid artery aneurysm. **(A)**, **(D)** Coronal view. **(B)**, **(E)** Sagittal view. **(C)**, **(F)** Three-dimensional rendering.

DSA may still be required for treatment planning in complex aneurysms that include branching points of large vessels. However, modern CTA techniques, including subtraction CTA and three-dimensional reconstructions, can provide appropriate imaging even in complex cases (Figure 2). DSA is definitely required in patients with diffuse nonperimesencephalic SAH and negative initial CTA. In this specific situation, DSA should be performed to rule out small aneurysms [7,14], and may be repeated within 2 to 6 weeks if the initial DSA is negative [24].

MRA is a possible alternative to CTA or DSA for aneurysm detection, especially in patients with iodine allergy. This modality does not expose the patients to radiation, and does not necessarily require gadolinium contrast injection. A recent published high-quality meta-analysis (Additional file 1: Table S2) including 12 studies (960 patients assessed, 772 aneurysms) compared MRA with DSA as the gold standard for aneurysm detection. Most studies used time-of-flight sequences, which do not require contrast. The pooled MRA sensitivity and specificity were 95% (95% CI, 89 to 98%) and 89% (95% CI, 80 to 95%), respectively [25]. The main disadvantages of this modality are availability, costs, and the time consumed to acquire the images.

## Angiographic vasospasm and delayed cerebral ischaemia

ACV is the narrowing of intracranial arteries detected by vascular imaging, while DCI is a syndrome defined as a focal neurological impairment, a decrease of at least 2 points on the Glasgow Coma Scale, or an increase of two points on the National Institutes of Health Stroke Scale that is not explained by other causes, such as hydrocephalus, rebleeding, or medical complications [26,27]. DCI is associated with higher rates of cerebral infarction, poor neurological outcome, and increased mortality [3,4,28,29].

Historically, DCI occurring within 2 weeks of aneurysm rupture has been attributed to ACV. However, one-half of the patients with severe ACV (>50% decrease in vessel diameter) will not develop DCI and up to 20% of patients without ACV (0 to 25% decrease in vessel diameter) will develop symptoms, which suggests that other factors besides ACV must play a role in the aetiology of DCI [30,31].

### Transcranial Doppler ultrasonography

**Figure 3:**
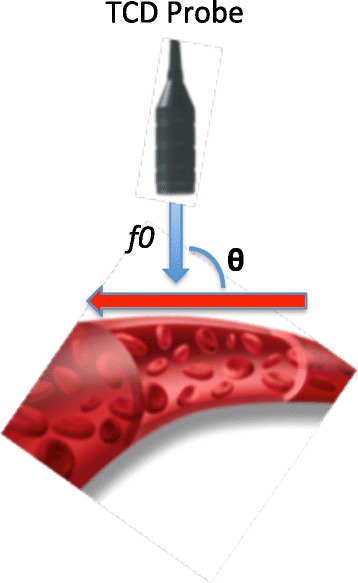
**Technical aspects.** Transcranial Doppler ultrasonography (TCD) probe emits low-frequency (2 MHz), pulsed waves that cross the skull at specific points called acoustic windows. The emitted waves are reflected back from the moving red blood cells at an altered frequency *fe*. The Doppler effect (*fe*) is the difference in frequency between the transmitted wave *f0* and the received wave *fe*: *fd = fe – f0.* The red blood cells’ velocity can then be calculated according to the following mathematic equation: *V = c* × *fd / 2* × *f0* × *cosθ.* θ is the angle formed by the ultrasound beam and the blood flow. Ideally this angle should approach 0° so that the ultrasound beam is parallel to the blood flow (cos0° = 1). The higher this angle, the lower the velocity read by the machine. If the ultrasound beam is perpendicular to the blood flow, no velocity will be detected (cos90° = 0). This concept is fundamental to the performance and interpretation of TCD results. The velocities can be underestimated, never overestimated.

**Figure 4:**
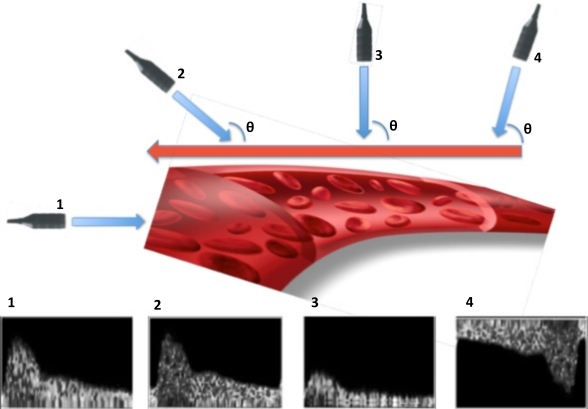
**Effect of the angle between the ultrasound beam and blood flow.** (1) Ultrasound beam is parallel to blood flow, acquiring an optimal, high-frequency Doppler signal. (2) Ultrasound beam forms an angle <20°, leading to a good signal. (3) Ultrasound beam is almost at 90°, resulting in a damped waveform. (4) Blood flow direction is away from ultrasound beam, which results in a negative Doppler signal.

Transcranial Doppler ultrasonography (TCD) is a non-invasive, reproducible, widely available technique that is commonly used to screen for ACV (Figures 3 and 4) [3,32,33]. This technique is based on the principle that the velocity of blood in a given artery is inversely proportional to the cross-sectional area of the artery. The mean middle cerebral artery (MCA) flow velocity is directly correlated to the degree of ACV by DSA [34] (Tables 1 and 2). A mean MCA flow velocity ≥120 cm/second or an increase ≥50 cm/second over a 24-hour period is consistent with ACV, and a mean MCA flow velocity ≥200 cm/second is diagnostic of severe ACV [13,33].

**Table 1:** **Transcranial Doppler characteristics**

**Artery**	**Window**	**Depth (mm)**	**Direction of flow**	**Mean flow velocity (cm/second)**
Middle cerebral	Temporal	40 to 60	Toward probe	55 ± 12
Anterior cerebral	Temporal	60 to 85	Away from probe	50 ± 11
Posterior cerebral	Temporal	60 to 70	Toward	40 ± 10
Extracranial internal carotid	Submandibular	50	Away	28 ± 17
Vertebral	Occipital	60 to 80	Away	38 ± 10
Basilar	Occipital	80 to 120	Away	41 ± 10

**Table 2:** **Middle cerebral artery criteria of angiographic vasospasm by transcranial Doppler ultrasonography**

	**Mean flow velocity (cm/second)**	**Hemispheric ratio (Lindegaard ratio)**
Normal values	35 to 89	<3
Above normal values	90 to 119	–
Mild vasospasm	120 to 150	3 to 4
Moderate	150 to 199	4 to 5.9
Severe	≥200	≥6

The Lindegaard ratio (the ratio of MCA to extracranial portion of internal carotid artery mean flow velocity) is a useful way to differentiate ACV from cerebral hyperaemia (Table 2). Lindegaard ratio <3 is considered normal, while a ratio ≥6 indicates severe ACV [33].

Several limitations of this technique should be mentioned: TCD is time consuming, is highly operator dependent, and requires experienced personnel to ensure consistent recordings. Up to 10% of patients, especially older women, do not demonstrate adequate acoustic windows [33]. More importantly, the accuracy for arteries other than the MCA is very poor. A high-quality meta-analysis [34] and a consensus statement [33] suggested that TCD is a useful tool to rule in vasospasm in the MCA, with high specificity and positive predictive value of 99% and 97%, respectively. Other studies have reported high specificity (94 to 100%) and variable sensitivity (39 to 96%) when TCD was compared to DSA in the MCA [33]. Negative results should be interpreted with caution because they may represent a false negative result. Transcranial Doppler assessment of arteries other than the MCA is less useful, because of the low sensitivity and specificity [32].

### Multimodal computed tomography (NCCT + CTA + computed tomography perfusion)

**Figure 5:**
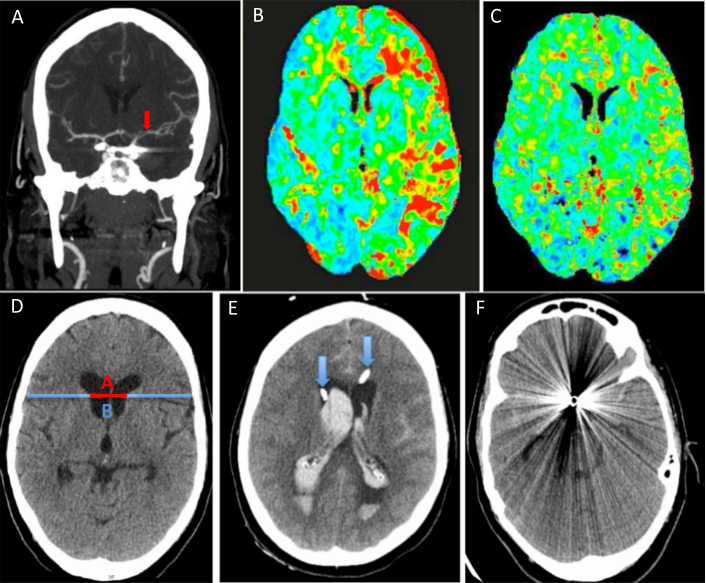
**Multimodal computed tomography. (A)** Computed tomography (CT) angiography (coronal plane) shows severe angiographic vasospasm in the left middle cerebral artery (MCA; red arrow). **(B)** CT perfusion displays increased mean transit time (increased shades of red in the left hemisphere) due to MCA vasospasm. **(C)** Same patient, CT perfusion repeated after 48 hours of haemodynamic augmentation, which shows resolution of perfusion changes. **(D) N**oncontrast computed tomography (NCCT) showing the measurement of bicaudate index (A/B) as proposed by van Gijn and colleagues [98]. **(E)** NCCT shows large volume of intraventricular haemorrhage. The tip of the bilateral external ventriculostomy drains can be seen in the anterior horn of the lateral ventricles (blue arrows). **(F)** NCCT quality severely compromised by artefacts generated by the metallic colis.

DCI is an exclusion diagnosis and can only be confirmed by imaging. Multimodal CT is frequently applied as a differential diagnostic tool at the moment of delayed neurological deterioration. NCCT is useful to exclude other causes of delayed neurological deterioration, such as hydrocephalus, rebleeding, or cerebral oedema. CTA is a good non-invasive alternative to DSA for diagnosing ACV (Figure 5A) [32]. Both techniques have a high degree of correlation for proximal ACV (agreement between 92 and 95%) [35,36]. However, the ability to monitor regional cerebral blood flow (CBF) and to assess global and regional cerebral perfusion is the main recent advance in DCI monitoring. Computed tomography perfusion (CTP) can be performed as a component of multimodal CT scanning (Figure 5B, C). The combination of CTA/CTP is more sensitive for ACV than TCD [33] and DSA [37].

Two high-quality meta-analyses that were recently published included 15 studies with 915 patients [38,39]. The first meta-analysis included studies that defined DCI as: clinical deterioration not explained by other causes; cerebral infarction on follow-up CT or MRI; and functional disability related to DCI. The results showed that SAH patients with perfusion changes measured by CTP had 23 times higher odds of having DCI than patients with normal CTP parameters (odds ratio, 23.14; 95% CI, 5.87 to 91.19). The pooled sensitivity and specificity of CTP for DCI were 0.84 (95% CI, 0.7 to 0.95) and 0.77 (95% CI, 0.66 to 0.82), respectively [40–45].

The second meta-analysis included original prospective and retrospective articles that reported data on the relationship between DCI (defined by clinical symptoms) and CTP. Perfusion parameters (CBF, mean transit time, time to peak, cerebral blood volume) were measured by either a quantitative method (comparing perfusion parameters in a given part of the brain with the same contralateral area) or a qualitative method (visual inspection of affected hemisphere compared with the contralateral side). The included studies were divided into two categories: CTP performed within 72 hours of haemorrhage (CTP as a predictor of DCI), and CTP performed between days 4 and 14 after SAH (CTP as a diagnostic tool for DCI). Early CTP parameters (<72 hours of SAH) were not predictive of DCI, but an increased mean transit time (pooled mean difference, 1.5 seconds; 95% CI, 0.9 to 2.2) or decreased CBF (difference, −11.9 ml/100 g/minute; 95% CI, −15.2 to −8.6) in the CTP performed between day 4 and day 14 was diagnostic for DCI [41,44,46–54].

In summary, the use of multimodal CT may be useful in the differential diagnosis of delayed neurological deterioration. Mean transit time and CBF values can distinguish between patients with and without DCI. However, the absolute thresholds may differ depending on the scanner and the post-processing methods applied.

## Short-term and long-term follow-up imaging after aneurysm treatment

### Intraoperative angiography

Perioperative imaging is useful to detect complications during and immediately after surgical clipping. Tang and colleagues evaluated the use of intraoperative angiography (IOA) performed during 517 consecutive craniotomies for aneurysm clipping [55]. In 12.4% of the cases, IOA findings resulted in surgical treatment change. Residual aneurysm (47%) and vessel flow compromise (44%) were the two most common causes of treatment change prompted by IOA. Aneurysms located in the internal carotid artery or giant aneurysms (>24 mm) were the two factors independently associated with treatment revision. Other case series have described similar results [56].

### Long-term follow-up

**Figure 6:**
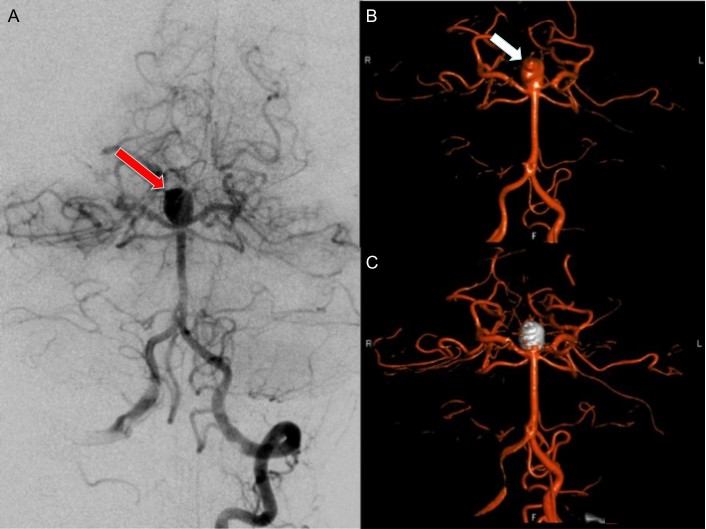
**Digital subtraction angiography. (A)** Selective left vertebral artery injection shows a basilar tip aneurysm (red arrow). **(B)** The same basilar tip aneurysm (white arrow), now appreciated through a three-dimensional rendering. **(C)** The gray structure is the mass of coils packing the previous basilar tip aneurysm. Final results show a complete obliteration of the aneurysm’s sac after the endovascular coiling.

The international SAH trial of neurosurgical clipping versus endovascular coiling (International Subarachnoid Aneurysm Trial) followed patients with SAH for a mean period of 9 years after treatment [57]. Patients treated by endovascular coiling have a 23% relative risk reduction and a 7.4% absolute risk reduction in death and dependence [58]; however, this is at a cost of significantly higher rates of rebleeding (2.5% vs. 1%) and a need for late retreatment (6.9 times higher rate) [59]. The main determinant of postoperative rebleeding risk is whether or not the aneurysm is completely excluded from the cerebral circulation (Figure 6).

After endovascular treatment, the mean time to late retreatment (that is, >3 months after coiling or 1 month after clipping) was 20.7 months (range, 3 to 80 months) versus 5.7 months in the clipped group. Younger patients (<40 years old), larger aneurysms (≥11 mm), and incomplete occlusion (Additional file 1: Table S3) are the main risk factors for retreatment after coiling [59,60]. Long-term follow-up imaging is therefore usually required to detect aneurysm recurrences, especially after coiling [59].

**Figure 7:**
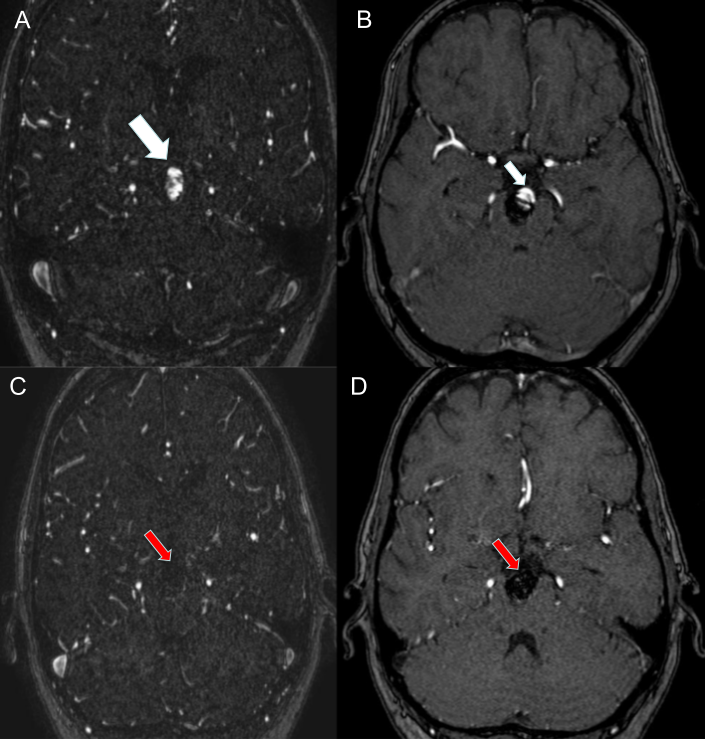
**Magnetic resonance angiography. (A)** Contrast-enhanced (CE) magnetic resonance angiography (MRA) sequence showing recanalisation of a previously coiled basilar tip aneurysm (large white arrow). **(B)** Time-of-flight MRA sequence revealing the refilling of the same basilar tip aneurysm (small white arrow). **(C)** CE-MRA and **(D)** time-of-flight MRA sequences after retreatment. No additional filling of the aneurysm is seen (red arrows).

MRA may be an attractive alternative to DSA in the setting of post-coiling follow-up (Figure 7). MRA is non-invasive, usually does not require contrast, and does not expose the patient to radiation.

van Amerongen and colleagues performed a high-quality systematic review and meta-analysis of 26 studies comparing MRA with DSA for follow-up of coiled aneurysms [61]. Both time-of-flight and contrast-enhanced MRA were highly accurate. The pooled time-of-flight MRA sensitivity and specificity were 86% and 84%, respectively; similarly contrast-enhanced MRA had 86% sensitivity and 89% specificity.

Even though aneurysm recurrence after clipping is less frequent, imaging follow-up is still required. Multiple studies comparing postoperative CTA versus DSA have been published with similar results [62–68]. Multidetector CTA is highly sensitive and specific for residual aneurysm detection and parent vessel occlusion, and might be a valuable diagnostic alternative to DSA. However, DSA might still be required for aneurysm with multiple clips [67,69], for neck remnant detection [68], or for aneurysms located in the posterior circulation [63].

The main concern with the use of postoperative CTA for aneurysm follow-up is the image quality, which can be compromised by clip-generated artefacts (Figure 5F) [64]. The factors associated with CTA image quality include the clip material (cobalt is worse than titanium alloy clips) [69,70], clip orientation (within the scan plane), and the number of clips on the same aneurysm [64,69]. Metal artefact reduction algorithms using flat-panel detector CT [71,72], subtraction three-dimensional CTA with the orbital synchronised helical scan technique [65], and advanced 3 T MRI imaging techniques [73] are some of the new modalities applied to reduce artefacts generated by the metallic clips, which can in turn improve the quality of the image.

In summary, patients treated by either surgical clipping or endovascular coiling should be followed after aneurysm occlusion [59]. Ideally, IOA should be performed to detect complications during surgical clipping. After endovascular coiling, the postoperative surveillance by either DSA or MRA is recommended. MRA with either time-of-flight or contrast-enhanced sequences may be the modality of choice. Clipped aneurysms can be followed by the use of CTA, but DSA should be performed if clip artefacts prevent adequate image interpretation. The time interval between images and duration of follow-up remain unclear.

## Future directions

Several emerging imaging modalities are currently available for research purposes, such as magnetic resonance-based images (including functional MRI) and positron emission tomography (PET). In the near future, these evolving fields of neuroimaging may serve as important tools when choosing treatment options and predicting functional and cognitive recovery after SAH.

### Magnetic resonance-based modalities

#### Structural magnetic resonance imaging

Structural damage caused by the primary haemorrhage, treatment, or DCI is not infrequent after SAH, and can ultimately lead to neuropsychological deficits and cognitive impairment. MRI can identify these structural changes [74,75]. Parenchymal high-signal intensity lesions detected on T2-weighted and intermediate-weighted images, and low signal intensity on three-dimensional T1-weighted images can represent a variety of aetiologies such as ischaemic lesions, residual haematoma, retraction injury due to surgical manipulation and instrumentation, lesions associated with external ventricular drains (Figure 5E) or permanent shunt placement, and previous infarctions. These high-signal intensity lesions are more frequent after surgical clipping and the lesion volume correlates with neuropsychological test performance [76].

#### Magnetic resonance imaging morphometry

The measurement of temporomesial volume by volumetric MRI (the protocol includes T2-weighted and proton density-weighted images, MRA, and three-dimensional T1-weighted sequences) [77], especially the hippocampal formation, can provide important information on cognitive performance [78]. Lesions affecting the bilateral medial temporal lobes typically lead to severe memory deficits. Unilateral lesions in the dominant hemisphere can cause deficits in verbal memory, while unilateral lesions in the nondominant hemisphere can cause visual–spatial memory loss. Aneurysmal SAH was shown to be associated with temporomesial atrophy, especially after surgical clipping [76]. Temporomesial volume loss is correlated with neuropsychological impairment, which includes memory deficits [78].

Bendel and colleagues examined the impact of brain structure volume changes on neurocognitive performance by evaluating the MRI scans of 138 patients that were randomly assigned to surgical versus endovascular treatment 1 year after the haemorrhage. Reduced hippocampal volume correlated with poor score on several measures of cognitive function such as visual memory, attention, flexibility of mental processing, intellectual ability, and psychomotor speed [78]. These deficits are more pronounced in patients who have combined decreased hippocampal volume and frontal lobe lesions, and are more common after surgical clipping than endovascular coiling [79].

The same group described the relationship of global cerebral atrophy and cognitive deficit after SAH [80]. Seventy-six patients (1 year after haemorrhage) and 30 healthy controls underwent MRI (T2-weighted and proton attenuation-weighted images, and three-dimensional T1-weighted images needed for volumetry). The cerebrospinal fluid/total intracranial volume ratio was quantified by statistical parametrical mapping, and was statistically higher in SAH patients and correlated with clinical outcome (that is, Glasgow outcome scale) and cognitive deficit.

#### Diffusion-weighted imaging

Diffusion-weighted imaging identifies tissues at risk for ischaemia by detecting restriction to water diffusion. Diffusion-weighted imaging is a potent instrument for the detection of early brain injury after SAH [81], presenting higher sensitivity compared to NCCT [82,83]. Diffusion-weighted imaging may predict functional outcome after poor-grade SAH [84,85]. In a very elegant study, Sato and colleagues included 38 poor-grade SAH patients that were treated aggressively. Lesions >10 mm on diffusion-weighted imaging scans performed within 24 hours of haemorrhage were predictive of poor outcome (severe disability, vegetative state or death). The authors attributed these lesions to primary brain damage (that is, haemorrhage) [84]. This hypothesis of early brain ischaemia has been supported by recent work that showed early infarct volume measured by diffusion-weighted imaging/apparent diffusion coefficient correlated with death or severe disability (adjusted odds ratio, 1.7; 95% CI, 1.0 to 3.2; *P* = 0.066) [86].

#### Functional magnetic resonance imaging

Functional MRI is a neuroimaging modality that measures brain activity by detecting changes in CBF. The modality was first described by Seiji Ogawa, using what he called blood oxygen level-dependent contrast [87]. This technique assumes that CBF is coupled with neuronal activation; therefore, CBF increases towards a given brain region that is in use.

Functional MRI represents a possible step towards our capability of prognosticating patients suffering from acute brain injury, including ischaemic stroke [88], intracerebral haemorrhage [89], and SAH [90]. This modality has a potential advantage over structural modalities by being able to assess brain function in real time. However, its use remains largely experimental.

The functional MRI literature in the SAH population is very scant. In a single prospective study, 11 SAH patients and 10 healthy subjects underwent functional MRI while performing verbal working memory tasks. Patients performed worse than healthy individuals (percent correct 82.9% vs. 97.5%, *P* <0.003). The memory impairment was accompanied by a relatively increased blood oxygen level-dependent signal in widespread bilateral cortical areas. The authors hypothesised that ‘deficits in verbal working memory following recovery from SAH are accompanied by widespread differences in hemodynamic correlates of neural activity. These differences are discussed with respect to the immediate and delayed focal and global brain damage that can occur following SAH, and the possibility that this damage induces subcortical disconnection and subsequent decreased efficiency in neural processing’ [90].

### Positron emission tomography

PET is a modality that maps perfusion and physiological activity by the use of exogenous radioactive compounds that are injected systemically. PET records and interpolates anatomical regions of the brain through detection of electromagnetic radiation; it also measures global and regional CBF, and also the oxygen delivery to cerebral tissues, which are important endpoints for DCI monitoring [91–94].

Over the last decade, a group from the Washington University School of Medicine led by Dr Diringer has conducted a series of experiments to determine the optimal haemodynamic intervention that would improve cerebral oxygen delivery in a setting of DCI. In the first experiment, the authors examined the effects of a normal saline fluid bolus on regional and global CBF, measured by PET. Six SAH patients who developed DCI underwent PET scanning before and after a normal saline bolus of 15 ml/kg administered over 1 hour. The fluid bolus was associated with increased CBF in hypoperfused regions of the brain [95]. In the second experiment, eight anaemic (<10 g/dl) SAH patients underwent PET scanning before and after the transfusion of 1 unit of red blood cells. Blood transfusion was associated with increased cerebral oxygen delivery in areas of ischaemia, reduction of oxygen extraction fraction, and no change in global CBF [96].

In the last published experiment, the authors compared the effect of a saline fluid bolus of 15 ml/kg, of induced hypertension by increasing mean arterial pressure by 25%, and of the transfusion of 1 unit of red blood cells [97]. Regional and global CBF, as well as oxygen delivery, were measured by PET imaging in 38 SAH patients that developed DCI. All three methods increased regional oxygen delivery from baseline, but red blood cell transfusion to anaemic patients (haemoglobin <9.0 g/dl) performed better than both hypertension and volume loading as a haemodynamic intervention in reducing the ischaemic burden.

Despite the interesting conclusions drawn from those studies, the utility of PET remains limited to research. The required isotopes are very expensive and restricted to major centres, thus limiting the technique’s clinical applications.

## Conclusion

Neuroimaging is a fundamental component in the management of patients suffering from SAH. NCCT might be sufficient to rule out SAH if performed within 6 hours of headache onset. However, patients presenting beyond the 6-hour mark with symptoms suspicious for SAH and negative NCCT should undergo LP. The combinations of NCCT and CTA or MRI and MRA are at least as sensible as the classic NCCT and LP approach, and can be used to rule out SAH. CTA is slowly replacing DSA as the first-line technique for the diagnosis and treatment planning of cerebral aneurysms, but DSA is still required in patients with diffuse SAH and initially negative CTA. The modern concept of DCI monitoring is shifting from modalities used to detect angiographic vasospasm to techniques focusing on brain perfusion, such as CTP. Lastly, new modalities applied to assess cerebral physiological, functional, and cognitive outcome after SAH are rapidly evolving; however, their use remains experimental.

## Additional file

Additional file 1:
**The following additional data are available with the online version of this paper. **Additional file 1 is Table S1 presenting PubMed search criteria, Table S2 presenting methodological quality of meta-analyses, and Table S3 presenting the degree of aneurysm occlusion.

## Abbreviations

ACV: Angiographic cerebral vasospasm
CBF: Cerebral blood flow
CI: Confidence interval
CT: Computed tomography
CTA: Computed tomography angiography
CTP: Computed tomography perfusion
DCI: Delayed cerebral ischaemia
DSA: Digital subtraction angiography
ED: Emergency department
IOA: Intraoperative angiography
LP: Lumbar puncture
MCA: Middle cerebral artery
MRA: Magnetic resonance angiography
MRI: Magnetic resonance imaging
NCCT: Noncontrast computed tomography
PET: Positron emission tomography
SAH: Subarachnoid haemorrhage
TCD: Transcranial Doppler ultrasonography
