# Supplementary material for: Aneurysmal subarachnoid haemorrhage from a neuroimaging perspective
Source: Crit Care. 2014 Nov 13;18(6):557. doi: 10.1186/s13054-014-0557-2 (PMC4331293; doi:10.1186/s13054-014-0557-2)
Supplement: Additional file 1: — The following additional data are available with the online version of this paper. Additional file 1 is Table S1 presenting PubMed search criteria, Table S2 presenting methodological quality of meta-analyses, and Table S3 presenting the degree of aneurysm occlusion. [file 13054_2014_557_MOESM1_ESM.docx]

**Additional file 1**

**Table S1 – Pubmed search criteria**

| **Pubmed** |
| --- |
| **1) SAH & synonyms:**  SAH [Title/Abstract] OR “subarachnoid hemorrhage” [Title/Abstract] OR “subarachnoid haemorrhage” [Title/Abstract] OR ASAH [Title/Abstract]  **2) CT & synonyms AND SAH & synonyms:**  (CT [Title/Abstract] OR “computed tomography” [Title/Abstract]) AND (SAH [Title/Abstract] OR “subarachnoid hemorrhage” [Title/Abstract] OR “subarachnoid haemorrhage” [Title/Abstract] OR ASAH [Title/Abstract])  **3) MRI & synonyms AND SAH & synonyms:**  (MRI [Title/Abstract] OR “magnetic resonance imaging” [Title/Abstract] OR "functional MRI" [Title/Abstract] OR fMRI [Title/Abstract] OR "diffusion weighted imaging" [Title/Abstract] OR DWI [Title/Abstract]) AND (SAH [Title/Abstract] OR “subarachnoid hemorrhage” [Title/Abstract] OR “subarachnoid haemorrhage” [Title/Abstract] OR ASAH [Title/Abstract])  **4) DCI OR vasospasm & synonyms:**  DCI [Title/Abstract] OR “delayed cerebral ischemia” [Title/Abstract] OR “cerebral ischemia” [Title/Abstract] OR “cerebral infarction” [Title/Abstract] OR OR stroke [Title/Abstract] OR vasospasm [Title/Abstract] OR “cerebral vasospasm” [Title/Abstract] OR “angiographic vasospasm” [Title/Abstract] OR “angiographic cerebral vasospasm” [Title/Abstract] |

**Table S2 - The methodological quality of meta-analyses assessed by AMSTAR**

| **Author** | **Year** | **Title** | **Reference** | **Topic** | **Patients and Studies Included** | **Major Findings** | **Level of Evidence** | **AMSTAR Grade** |
| --- | --- | --- | --- | --- | --- | --- | --- | --- |
| Cloft HJ et al. | 1999 | Risk of cerebral angiography in patients with subarachnoid hemorrhage, cerebral aneurysm, and arteriovenous malformation: a meta-analysis. | Stroke. 1999 Feb;30(2):317-20. | Aneurysm Treatment | 3517 patients, from 3 prospective studies of complications in cerebral angiography | The risk of permanent neurological complication associated with cerebral angiography in patients with SAH, cerebral aneurysm, and AVM is low (0.07%). | B | 2 |
| Lysakowski et al. | 2001 | Transcranial Doppler versus angiography in patients with vasospasm due to a ruptured cerebral aneurysm: A systematic review. | Stroke. 2001 Oct;32(10):2292-8. | Vasospasm Monitoring | Twenty-six reports comparing TCD with angiography. | For the middle cerebral artery sensitivity was 67% (95% CI 48% to 87%), specificity was 99% (98% to 100%), positive predictive value (PPV) was 97% (95% to 98%), and negative predictive value (NPV) was 78% (65% to 91%), therefore TCD is not likely to indicate a spasm when angiography does not show one (high specificity), and TCD may be used to identify patients with a spasm (high PPV). For all other situations and arteries, there is either lack of evidence of accuracy or of any usefulness of TCD. | B | 8 |
| Kwee et al. | 2007 | MR angiography in the follow-up of intracranial aneurysms treated with Guglielmi detachable coils: systematic review and meta-analysis | Neuroradiology (2007) 49:703–713 | Aneurysm Treatment/Follow-up |  |  |  |  |
| Vergouwen et al. | 2011 | Lower incidence of cerebral infarction correlates with improved functional outcome after aneurysmal subarachnoid hemorrhage. | J Cereb Blood Flow Metab. 2011 Jul;31(7):1545-53. | Prognosis | 8552 patients, 24 randomized, double-blind, placebo-controlled trials. | Pharmaceutical treatments decreased both cerebral infarction and poor functional outcome after SAH. Cerebral infarction is associated with worse functional outcome. | A | 9 |
| Menke et al. | 2011 | Diagnosing cerebral aneurysms by computed tomographic angiography: meta-analysis. | Ann Neurol. 2011 Apr;69(4):646-54. | SAH diagnosis | 3643 patients, 45 studies | CT angiography has a high accuracy in diagnosing cerebral aneurysms, specifically when using modern multidetector CT. | A | 10 |
| Westerlaan et al. | 2011 | Intracranial aneurysms in patients with subarachnoid hemorrhage: CT angiography as a primary examination tool for diagnosis--systematic review and meta-analysis. | Radiology. 2011 Jan;258(1):134-45. | SAH diagnosis | 50 studies | Multidetector CT angiography can be used as a primary examination tool in the diagnostic work-up of patients with SAH. | A | 10 |
| Mir et al. | 2013 | CT Perfusion for Detection of Delayed Cerebral Ischemia in Aneurysmal Subarachnoid Hemorrhage: A Systematic Review and Meta-Analysis | AJNR Am J Neuroradiol. 2013 Dec 5. [Epub ahead of print] | CT perfusion and delayed cerebral ischemia | 345 patients, 6 cohort studies | Patients with aSAH with positive CTP test results were approximately 23 times more likely to experience delayed cerebral ischemia compared with patients with negative CTP test results. | B | 8 |
| van Amerongen et al. | 2013 | MRA Versus DSA for Follow-Up of Coiled Intracranial Aneurysms: A Meta-Analysis | AJNR Am J Neuroradiol. 2013 Sep 5. [Epub ahead of print] | Aneurysm Treatment/Follow-up | 26 studies | Both TOF-MRA and contrast-enhanced MRA are shown to be highly accurate for detection of any recanalization in intracranial aneurysms treated with endovascular coil occlusion. | B | 9 |
| Cremers et al. | 2014 | CT perfusion and delayed cerebral ischemia in aneurysmal subarachnoid hemorrhage: a systematic review and meta-analysis. | J Cereb Blood Flow Metab. 2014 Feb;34(2):200-7. | CT perfusion and delayed cerebral ischemia | 570 patients, 11 studies included | DCI was associated with a decreased CBF (pooled mean difference 11.9 mL/100 g per minute (95% confidence interval (CI): 15.2 to 8.6)) and an increased MTT (pooled mean difference 1.5 seconds (0.9–2.2)), but no difference was found when CTP was performed within 72h of hemorrhage. Therfore, CTP can be used in the diagnosis but not in the prediction of DCI. | B | 9 |
| Bakker et al. | 2014 | Repeat digital subtraction angiography after a negative baseline assessment in nonperimesencephalic subarachnoid hemorrhage: a pooled data meta-analysis. | J Neurosurg. 2014 Jan;120(1):99-103. | SAH diagnosis | 368 patients, 8 studies included | In 37 patients (10.0%, 95% CI 7.4%–13.6%), an aneurysm was detected using repeat DSA. Conclusion: repeat DSA is still warranted in patients with a diffuse nonperimesencephalic SAH and negative initial assessment. Timing is not clear, though. | B | 5 |
| Sailer et al. | 2014 | Diagnosing Intracranial Aneurysms With MR Angiography: A Systematic Review and Meta-analysis | Stroke. 2014;45:119-126 | SAH diagnosis | 960 patients, 12 studies | Pooled sensitivity of MRA was 95% (95% confidence interval, 89%–98%), and pooled specificity was 89% (95% confidence interval, 80%–95%). | B | 10 |

**Table S3 – Degree of aneurysm occlusion after treatment**

|  | Occlusion Grade 1 | Occlusion Grade 2 | Occlusion Grade 3 |
| --- | --- | --- | --- |
| Endovascular | Total occlusion, no contrast filling of the aneurysm sac | Subtotal occlusion, minor residual sac filling or neck remnant | Incomplete occlusion, substantial residual sac filling |
| Surgical Clipping | Total occlusion, no blood flow or contrast filling of the aneurysm sac | Incomplete occlusion (clipping or wrapping), any blood flow or contrast filling of the aneurysm sac |  |
